# Prevalence and Clinical Picture of Diamine Oxidase Gene Variants in Children and Adolescents with Attention Deficit Hyperactivity Disorder: A Pilot Study

**DOI:** 10.3390/jcm13061659

**Published:** 2024-03-14

**Authors:** Hilario Blasco-Fontecilla, Marcos Bella-Fernández, Ping Wang, Marina Martin-Moratinos, Chao Li

**Affiliations:** 1 UNIR-Itei & Health Sciences School, Universidad Internacional de La Rioja, 26004 Madrid, Spain; 2 Center of Biomedical Network Research on Mental Health (CIBERSAM), Carlos III Institute of Health, 28029 Madrid, Spain; 3 Service of Child and Adolescent Psychiatry, Puerta de Hierro University Hospital-Majadahonda, 28222 Madrid, Spain; marcosbellafernandez@gmail.com (M.B.-F.); mmmoratinos27@gmail.com (M.M.-M.); 4 Faculty of Psychology, Autonomous University of Madrid, 28049 Madrid, Spain; 5 Department of Psychology, Pontifical University of Comillas, 28049 Madrid, Spain; 6 Faculty of Medicine, Autonomous University of Madrid, 28029 Madrid, Spain; wpedith@gmail.com (P.W.); ericlimed@gmail.com (C.L.)

**Keywords:** DAO, ADHD, genetics, prevalence, intelligence

## Abstract

**Background:** Attention Deficit Hyperactivity Disorder (ADHD) is the most prevalent neurodevelopmental disorder worldwide. The diamine oxidase enzyme (DAO) is responsible for the histamine gastrointestinal degradation. Its deficient functioning may implicate an excess of histamine in the body. The excess of histamine (histamine intolerance, HIT) has been related with a growing number of diseases and pseudo-allergic symptomatology. However, data on the relationship between the DAO enzyme, HIT, and ADHD are lacking. The main objective of this pilot is to study the prevalence of the four most relevant SNP variants of the AOC1 gene affecting DAO enzyme functionality in a sample of patients diagnosed with ADHD attending child and adolescent mental health services. **Methods:** In a cohort of 303 participants, we measured the SNP variants of the AOC1 gene. **Results:** The prevalence of having at least one minor dysfunctional allele was 78.8%. No relationship between ADHD severity and DAO deficiency was found. However, some AOC1 gene variants associated with DAO deficiency were related to several meaningful medical comorbidities. Furthermore, we found a strong association between DAO activity and the intelligence quotient, particularly in working memory. **Conclusions:** Some SNP variants of the AOC1 gene associated with DAO deficiency are related to some medical comorbidities and cognitive dysfunction in ADHD children and adolescents. Studies including patients with other diagnoses and healthy controls and bigger samples are warranted to confirm our preliminary results.

## 1. Introduction

Attention Deficit Hyperactivity Disorder (ADHD) is the most prevalent neurodevelopmental disorder. ADHD is diagnosed in around 5% of children worldwide [1]. ADHD is characterized by age-inappropriate inattention, hyperactivity/impulsivity, or both. The treatment of choice is multimodal treatment. The most relevant drugs used to treat ADHD increase mainly dopamine (DA) and norepinephrine (NE), either pre- or post-synaptic [2]. Unfortunately, the current multimodal treatment is insufficient for the full recovery of patients with ADHD. Therefore, 40% of patients with ADHD will still be diagnosed during adulthood. Accordingly, novel treatment strategies are warranted.

The insufficient role of the current pharmacological treatments may be related to the fact that most of the pathophysiology and etiology of ADHD is based in the catecholamine system (DA and NE) [2]. Unfortunately, the potential role of histamine in the pathophysiology of ADHD has been neglected. This is unfortunate because histamine levels [3], as well as histamine-specific single-nucleotide polymorphisms (SNPs) variants [4], are associated with the development of cognitive disorders, including ADHD (see Carthy and Ellender [5] for a review). Furthermore, antihistamine use has been associated with the subsequent detection of ADHD [6,7]. This increased use of antihistamine drugs may be related to the increased risk of other diseases such as atopy [7,8,9], food allergies [10], and allergic rhinitis [11,12] in people with ADHD. This respiratory symptomatology could suggest an involvement of immunoglobulin E (IgE) [12], which, in turn, has also been linked to ADHD [13]. Furthermore, most of these disorders are usually included within a relatively unknown entity, histamine intolerance (HIT). A recent study postulated that histamine might be the missing link between ADHD and medical comorbidities usually included within Allergic Tension Fatigue Syndrome [14].

Histamine is an imidazole amine that is involved in local immune responses (related to leukocyte and eosinophil chemotaxis), the digestive system, and the central nervous system, where it acts as a neurotransmitter. Although our body synthesizes it, foods also contain varying amounts of histamine. Histamine is metabolized by two main enzymes: diamine oxidase (DAO) and histamine-N-methyltransferase, HNMT [15,16]. HNMT is responsible for intracellular histamine, whereas DAO metabolizes histamine extracellularly [17]. DAO has a higher expression than HNMT, being the main barrier for the intestinal absorption of histamine [17]. At least in the Caucasian population, the SNPs that can most directly cause DAO deficiency are rs10156191, rs1049742, rs1049793, and rs2052129 [18,19,20]. Furthermore, the role of DAO has usually been associated with body functions, whereas the activity of HNMT is basically reduced to the central nervous system (CNS).

DAO deficiency has been linked to migraines [21], celiac disease, gluten intolerance [22], and HIT, which is related to various cutaneous, respiratory, and gastrointestinal allergic symptoms, among others [23,24,25]. HIT is defined as an imbalance between accumulated histamine and its degradation capacity [15,17,23]. It is a non-immunologically mediated pathology characterized by decreased histamine degradation in some individuals [15,16]. Thus, some individuals are unable to adequately degrade ingested histamine, which subsequently results in hypersensitivity to normal or even low levels of histamine in food. The research on HIT has been growing over the last decade [15]. Interestingly, most of the disorders associated with DAO deficiency are also frequently reported in people diagnosed with ADHD. For instance, in a recent case–cohort study, the authors found an increased risk of migraine among ADHD probands compared with matched controls [26]. Moreover, there is increasing evidence about the relationship between ADHD and celiac disease [27].

The main objective of the present study is to explore the prevalence of the four SNP variants (rs10156191, rs1049742, rs1049793, and rs2052129) in the DAO gene typically associated with defective DAO functioning in children and adolescents previously diagnosed with ADHD. A second objective is to explore the association of these DAO polymorphisms and gender, cognitive skills, and comorbid medical conditions, particularly those related with allergic symptoms.

## 2. Materials and Methods

### 2.1. Sample and Measures

For this observational study, we obtained data from 303 children and adolescents with a primary diagnosis of ADHD following treatment at the Child and Adolescent Mental Health Services (CAMHS) at Puerta de Hierro University Hospital, Majadahonda (Madrid, Spain). In total, 65 patients had none of the 4 studied SNP variants associated with reduced DAO activity, whereas 238 patients had at least one of these variants. A post hoc power analysis with these sample sizes, assuming a confidence level of 0.95 and effect sizes of d = 0.4, gave a power estimation of 0.81.

The protocol included several sociodemographic and clinical data and some scales. For instance, we included information regarding all five Axes of the *Diagnostic and Statistical Manual, Fourth Edition* (DSM-IV) [28]. Furthermore, we included information, when available, regarding the *Wechsler Intelligence Scale for Children-Fourth Edition* (WISC-IV [29]), the most often used test of intelligence. The WISC-IV measures the intellectual quotient of children and adolescents aged between 6 and 16 years. The WISC-IV measures not only general cognitive ability but intellectual functioning in verbal comprehension (VC), perceptual reasoning (PR), working memory (WM), and processing speed (PS) [30]. Also, we included the Histamine Intolerance Clinic Questionnaire (HICQ), which is a non-validated questionnaire including 21 symptoms typical of HIT [25] grouped in four ambits: gastrointestinal, respiratory, dermatological, and cardiac. The HICQ was administered to assess a wide range of symptomatology, focusing on pseudo-allergic gastrointestinal and respiratory symptoms. Because the HICQ is a non-validated test, we also tested its internal validity through a parallel analysis (to estimate the number of dimensions) and the Cronbach’s alpha of each symptom group.

The participants were divided according to the DSM-IV ADHD subtypes: the ADHD combined/predominantly hyperactive subtype (ADHD) and ADHD predominantly inattentive (ADD).

This study was approved by the Ethics Committee for Clinical Research (CEIC) of the Puerta de Hierro University Hospital (code PI 163-21, approval date 19 February 2023). Written informed consent was obtained from all the participants.

### 2.2. AOC1 Variants Genotyping

We measured the four most relevant SNP variants of the AOC1 gene through the DAO-Test^®^ Genotyping Kit (DR Healthcare, Barcelona, Spain): Variant 1 (V1), p.Thr16Met (rs10156191); Variant 2 (V2), p.Ser332Phe (rs1049742); Variant 3 (V3), p.His664Asp (rs1049793); and Variant 4 (V4), c.691G>T (rs2052129). The gene encoding for the DAO/ABP1 protein is the AOC1 gene, for which the variants have been described, and some of them are associated with reduced levels of DAO activity. A deficiency of DAO activity is associated with a reduced histamine degradation capacity, which may result in histamine intolerance.

For instance, the T16M variant has also been associated with an increased risk of hypersensitivity to NSAIDs (non-steroidal anti-inflammatory drugs) and has been proposed as a biomarker of clinical response.

We chose these four variants because they are the ones that accumulate more research evidence regarding reduced DAO activity levels in Caucasian populations [31,32]. For each SNP variant, each patient can be homozygote (normal DAO functioning), heterozygote (mild DAO defective functioning), or homozygote (full DAO defective functioning) (see Table 1).

We collected saliva samples from oral mucosa by rubbing the inner side of both cheeks using a sterile cotton swab. Maintaining a clean mouth for 60 min before the sample collection was mandatory. The samples were kept at room temperature until they were sent to the laboratory (maximum time of 1 week). We used an automatic DNA isolation procedure (QIASymphony SP platform: QIAGEN, Hilden, Germany) with a QIASymphony DSP DNA Mini Kit (QIAGEN). The genotyping was performed with a Multiplex (Single-Nucleotide Primer Extension) SNPE followed by capillary electrophoresis in an ABI 3500 Genetic Analyzer (Thermofisher Scientific, Applied Biosystems, Waltham, MA, USA).

### 2.3. Statistical Analyses

The categorical variables were expressed as frequencies and percentages. The continuous variables were expressed as the mean and standard deviation (SD). We performed between-groups tests using Chi-square and Fischer’s exact tests for categorical variables and Mann–Whitney tests for numerical variables. In order to gain statistical power for some analyses, we merged the mild and severe DAO defective functioning, as the genotypes associated with a severe DAO defective functioning were rare. The intelligence quotient and cognitive skills are normally distributed; in these cases, we used parametrical tests (*t*-tests and ANOVAs) instead of non-parametrical tests. A statistical analysis was performed using SPSS 26.0. The level of statistical significance was set at *p* < 0.05, but we will comment on the findings at the *p* < 0.10 level if clinically meaningful.

## 3. Results

### 3.1. Characteristics of the Sample

Table 2 shows the sociodemographic and main clinical and neuropsychological variables split by gender. Regarding ethnicity, most of the sample was Caucasian, apart from 11 Latin American patients, 8 Asian patients, 2 Sub-Saharan African patients, 1 Arab patient, and 16 mixed patients. We found no statistically significant variables regarding gender.

### 3.2. DAO Deficiency

The prevalence of “genetic DAO deficiency” (the presence of at least one minor allele associated with DAO deficiency in the SPNs analyzed) in our sample was 78.5% (82.94% in females, and 77.06% in males, *p* = 0.397). We found no association between the DAO variants affected and gender, but we found that the Caucasian group seemed to have less DAO deficiency prevalence, particularly in variant 3 (see Table 3).

Furthermore, we found no relationship between either the genotype variants or number of affected variants with the ADHD subtype: 79.4% of the patients diagnosed with the ADHD combined/predominantly hyperactive subtype had at least one allele related to DAO deficiency compared with 77.5% in the ADD population (see Table 4).

**Figure 1:**
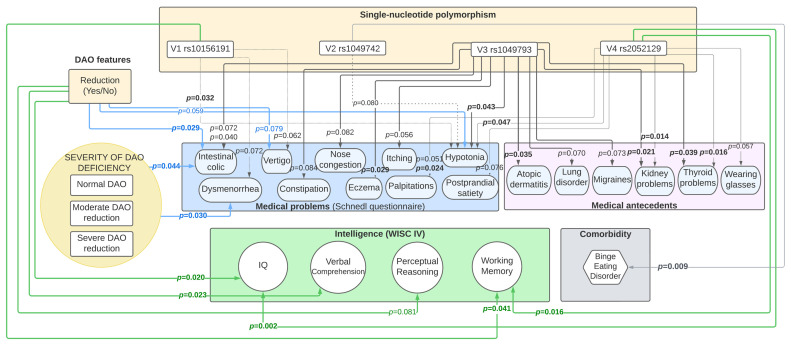
Relationship between DAO reduction and genotype variants and medical conditions (n = 303).

**Figure 2:**
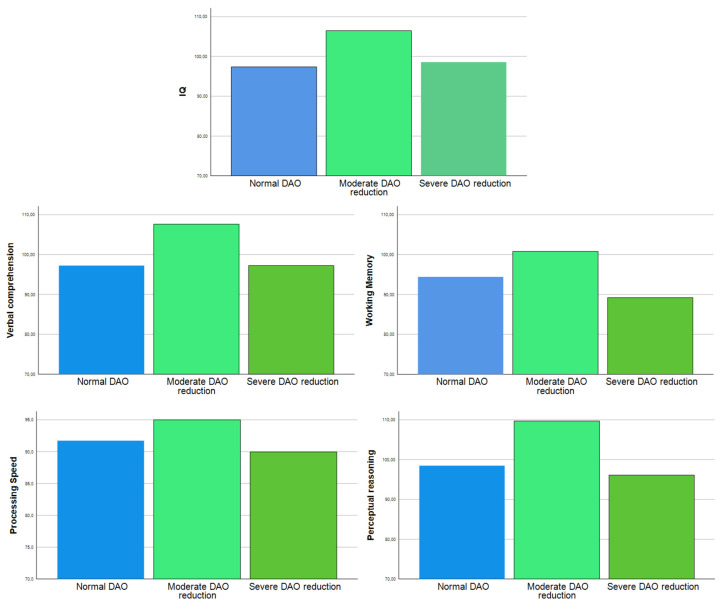
Relationship between DAO reduction severity and cognitive skills.

Figure 1 and Tables S1–S3 (Supplementary Material) show the statistically significant relationships between the DAO variants, antecedents of several medical diseases, and comorbid medical conditions. Figure 1 and Figure 2 and Table 5 and Table 6 show the statistically significant relationships between the DAO variants and IQ and cognitive skills. Regarding the associations between the AOC1 gene variants and several medical comorbidities, we focused on those not only statistically significant but also clinically meaningful to avoid commenting on spurious findings. For instance, we found a statistically interesting association between V4 (rs2052129) and antecedents of kidney problems. Thus, two patients had antecedents of kidney problems and the genetic genotype associated with DAO defective functioning, whereas there were no patients with the normal functioning genotype. However, given these figures (two patients), even if our results were statistically significant, they were clinically not. Accordingly, we gave little relevance to this finding. On the contrary, for instance, we found that variant 3 was marginally statistically associated with migraines (*p* = 0.073). However, this finding was clinically meaningful, as 20 patients (13.2%) had the defective (mild or severe) genotype in variant 3 (rs1049793).

Table 5 and Table 6 show the relationship between the four variants in the AOC1 gene and IQ. Unfortunately, we did not have data regarding the IQ and cognitive skills for all the participants; the number of participants we had data on is detailed in the tables. The working memory was compromised in patients with affected variants 1 and 4, while impaired IQ is only related with variations in variant 4. The participants with the TT genotype in variant 1 (rs10156191) had a working memory of 85.18 (18.04), whereas those with the CC genotype had 97.64 (17.09) (*p* = 0.041). Regarding variant 4 (rs2052129), those with the GG genotype had an IQ of 99.26 (18.53), whereas those with the GT genotype had 108.51 (21.39), and those with the TT genotype had 90.82 (11.37) (*p* = 0.002). Furthermore, this difference was mostly due to the differences in working memory: 96.19 (17.02) (GG genotype), 101.16 (17.98) (GT genotype), and 81.71 (8.73) (TT genotype) (*p* < 0.016). Variants 2 and 3 seem to have little effect on intelligence.

Furthermore, taken together, the number of variants and the phenotypic outcome of these variants did also influence IQ. We found a paradoxical effect of reduced DAO activity on increased general IQ and verbal comprehension. Furthermore, as shown in Figure 2, this increase in IQ and other cognitive skills seems to be related to an increase in the cognitive measures of patients with moderately reduced DAO activity.

### 3.3. Internal Validity of the HICQ Test

The parallel analysis suggested a four-dimensional factor structure, which is consistent with the four domains assessed: gastrointestinal, respiratory, dermatological, and cardiovascular. Subsequently, we estimated the reliability of each factor through Cronbach’s alpha (see Table 7). Alpha values above 0.7 are considered acceptable, although for short scales, as it is the case in some of the scales from the HICQ, this threshold may be softened. Anyway, all the factors except cardiovascular gave acceptable reliability values. In particular, the two most relevant factors for this research, gastrointestinal and respiratory symptoms, gave good alpha values.

## 4. Discussion

The present pilot study explored the relationship between four SNP variants (rs10156191, rs1049742, rs1049793, and rs2052129) of the DAO gene in children and adolescents diagnosed with ADHD. If confirmed in other samples by other authors, our results may have relevant clinical consequences. First, our sample confirmed the close association of ADHD with some comorbid conditions, particularly atopic diseases and traumatic injuries. Second, children and adolescents with ADHD in our sample had a high prevalence of alterations in the AOC1 gene that are associated with deficient functioning of the DAO enzyme. Third, we found no association between the different variants of the AOC1 gene and gender or ADHD subtype. However, we did find several statistically significant associations between the different AOC1 gene variants studied and antecedents and comorbidity with several medical comorbidities. Most of the statistically significant associations were related to pathologies encompassed within the HIT, giving further support to the association between the AOC1 gene and HIT. Furthermore, we found no relationship between either the genotype variants or number of affected variants with ADHD subtypes. But the most relevant finding was, somewhat, unexpected. We found a strong association between DAO activity and both IQ and an IQ parameter (WM). The participants with the TT genotype in variant 1 (rs10156191) had a WM of 85.18 (18.04), whereas those with the CC genotype had 97.64 (17.09) (*p* = 0.041). Regarding variant 4 (rs2052129), those with the GG genotype had an IQ of 99.26 (18.53), whereas those with the GT genotype had 108.51 (21.39), and those with the TT genotype had 90.82 (11.37) (*p* = 0.002). Furthermore, this difference was mostly due to the differences in WM: 96.19 (17.02) (GG genotype), 101.16 (17.98) (GT genotype), and 81.71 (8.73) (TT genotype).

As said above, the ADHD children and adolescents in our sample displayed the typical profile of these patients. For instance, around 90% of people worldwide are right-handed for many tasks [31]. However, right handedness was only 70% in our sample, in keeping with literature demonstrating a higher prevalence of atypical handedness in patients with ADHD compared to neurotypical individuals [32]. Furthermore, our sample displayed a high prevalence of typical medical comorbidities, such as trauma injury or atopic diseases. Regarding atopy, two recent meta-analytic studies concluded that atopic diseases were associated with ADHD [33,34] and another study confirmed a relationship between atopy and ADHD symptom severity [35].

The close relationship between atopy and ADHD may have something to do with the high prevalence of “genetic DAO deficiency”, an underlying cause of HIT, in our sample (78.5%; 82.94% in females, and 77.06% in males). Unfortunately, there is no previous literature regarding the prevalence of DAO deficiency in either ADHD samples or even the general population. The prevalence of genetic DAO deficiency is frequent in pathologies usually associated with HIT. For instance, a recent study reported that 88% of 100 patients with at least moderate lower urinary tract symptoms (an entity that the authors included within the HIT syndrome) had at least one minor defective allele of the DAO enzyme [36]. Another example is migraine. More than 85% of patients with migraine have DAO deficiency [37]. Finally, our results give some support to our hypothesis that DAO deficiency, an enzyme which metabolizes histamine extracellularly, might play a critical role in the pathophysiology of ADHD. Thus, decreased DAO activity might lead to an accumulation of histamine, which could explain both ADHD symptoms and comorbid disorders, such as atopy [14].

Furthermore, we found several interesting statistically significant associations between the different AOC1 gene variants and either antecedents or present comorbidity with several medical disorders. One of the clinically most relevant findings was, indeed, only marginally statistically significant: the association between variant 3 and migraine. A total of 20 ADHD patients (67% of those reporting migraine) had a genetic variant associated with DAO dysfunction compared with the 33% reporting migraine within the group of ADHD patients with a variant 3 genotype associated with normal DAO functioning. In 1971, Speer published a study relating to allergies and migraines [38], two common medical comorbidities among ADHD populations [39,40]. Furthermore, the Geschwind–Behan hypothesis study [41] suggests an association between left-handedness, which is more frequent in patients with ADHD as stated before, and immune diseases and migraine. The association between migraine and ADHD may probably be mediated by immune mechanisms where histamine metabolism, and therefore the DAO enzyme, is critical. Indeed, a recent review stressed that histamine is core in migraine pathogenesis via an inflammation pathway [42]. In another study, a close relationship between DAO deficiency and non-celiac gluten sensitivity (NCGS), and the most severe migraine symptomatology, was reported [22]. Furthermore, genetic DAO deficiency is related to migraine [21]. In another study, more than 85% of patients diagnosed with migraine displayed DAO deficiency [38].

We also found a clinically and statistically significant association between intestinal colic and DAO genetic variant 3 (rs1049793). In one study, 53% of the patients with HIT had intestinal colic [25]. Another interesting finding was the association between palpitations and DAO variant 4. We found that 64% of the ADHD patients with the defective variant had palpitations. In one study, 47% of the patients with HIT had palpitations [25]. To sum up, our findings are in keeping with the literature linking DAO deficiency with migraine and other medical disorders typically included within the HIT picture. Given that our sample was composed of patients with a primary diagnosis of ADHD, that most of them displayed at least one AOC1 variant related to DAO deficiency, and that some medications approved for the treatment of ADHD (i.e., lisdexamfetamine dimesylate) may improve DAO activity [43], thus helping to decrease blood histamine levels, we may conclude that some medications for ADHD may help to reduce both ADHD and HIT symptoms by killing two birds with one shot [14]. Another interesting finding was between variant 2 (rs1049742) and binge eating disorder. Unfortunately, we did not find literature to contrast this finding.

But the most relevant finding was, somewhat, unexpected and puzzling. We found a strong association between the allele of variants 1 (rs10156191) and 4 (rs2052129) of the AOC1 gene associated with defective DAO activity and WM. Furthermore, impaired IQ was only related with the defective alleles in variant 4. On the other hand, the number of variants and the phenotypic outcome of these variants did also influence IQ. We found a paradoxical effect of reduced DAO activity on increased general IQ and verbal comprehension. Furthermore, patients with at least one allele associated with DAO deficiency had a higher IQ. The ones with a higher IQ were those with four defective alleles, followed by those with just one defective allele. Finally, a higher IQ was associated with the genetic profile associated with mild, but not severe, DAO deficiency. To sum up, it seems that (1) the heterozygosity of variant 4 related to mild DAO deficiency was associated with a higher IQ; (2) the homozygosity of alleles within variants 1 and 4 related to severe DAO deficiency was associated with a lower IQ and, particularly, a much lower WM; and (3) ADHD children and adolescents with a higher IQ were those with four defective alleles, followed by those with just one defective allele. But how can these findings be explained?

The reality is that, despite histaminergic neurons being key for regulating learning, memory, locomotion, circadian rhythms, and feeding, among others [44], there is virtually no information regarding either the association between histamine and intelligence or between DAO and intelligence. Accordingly, we may only speculate about the reasons behind our finding. Histamine in the CNS is involved in learning and memory, and treatment with antihistamines characteristically impairs learning and memory (see [14] for a review of the role of each histamine receptor in the brain). A basic study demonstrated the critical role of histamine in long-term memory by providing the brain with the compensatory plasticity necessary to ensure memorization of emotionally salient events when one brain structure is compromised [45]. In another study with mice, the authors suggested that H1 receptor (H1R) deficiency was associated with pronounced deficits in hippocampus-dependent spatial learning and memory. They also provided evidence that H1R deficiency led to reduced neurogenesis [46].

Furthermore, the role of the DAO enzyme in the brain should theoretically be less relevant than the one of the HNMT enzyme. Indeed, two novel mutations in the human HNMT gene (G179A and T632C) impairing its enzymatic activity had been associated with intellectual disability [47]. However, as explained elsewhere [14], blood histamine does not pass the blood–brain barrier (BBB), and therefore, DAO deficiency should not influence brain functioning. However, the BBB is permeable to histamine during development. Accordingly, a DAO deficiency may influence some processes in the brain, such as learning and memory, and the development of some disorders such as ADHD by allowing for the permeability of histamine into the central nervous system during critical developmental periods.

## 5. Conclusions, Limitations, and Future Directions

In this pilot study, we have reported compelling evidence suggesting that (1) children and adolescents with ADHD have a very elevated prevalence (78.8%, nearly four out of five) of having at least one AOC1 gene allele associated with DAO deficiency. However, we cannot conclude that our findings are generalizable to ADHD as we lack information regarding the prevalence of the defective AOC1 gene alleles associated with DAO deficiency either in patients with other mental disorders or in the general population. In addition, we cannot compare our data with other studies because, to our knowledge, there is not another study exploring the ACO1 gene alleles in ADHD populations. (2) AOC1 gene variants associated with DAO deficiency were related to several meaningful medical comorbidities, i.e., the association between variant 3 [p.His664Asp (rs1049793)] and either migraine or intestinal colic or the association between the AOC1 gene variant 4 [c.691G>T (rs2052129)] and palpitations. (3) Some AOC1 gene variants associated with DAO deficiency may influence the IQ and particularly one cognitive parameter, WM. Thus, the heterozygosity of the AOC1 gene variant 4 [c.691G>T (rs2052129) related to mild DAO deficiency was associated with a higher IQ. On the contrary, the homozygosity of the alleles of both variant 1 [p.Thr16Met (rs10156191)] and variant 4 [c.691G>T (rs2052129)], which are related to severe DAO deficiency, was associated with a lower IQ and, particularly, a much lower WM. In any case, the correlations reported between the genetic variants and WM profiles require comparisons with other patient populations to draw more meaningful conclusions. (4) ADHD children and adolescents with a higher IQ were those with four defective alleles for DAO deficiency, followed by those with just one defective allele.

The major limitation of the present study was sample size. Even with a sample of 300 patients, we found several trends, particularly on the relationship between medical comorbidities and the AOC1 gene variants, that were not statistically significant. In any case, most of the statistically significant associations were related to pathologies encompassed within the HIT picture, giving further support to the association between the AOC1 gene and HIT. Another limitation was that, given the cross-sectional nature of this study, we could not make causal statements. Thus, a generalization of these results to the whole population of patients with ADHD should be made cautiously. Another limitation was the scarcity of the literature addressing critical areas of this study. For instance, to date, there is not a single study about the prevalence of AOC1 gene variants in the general population. For instance, if most of the population has at least one AOC1 gene variant associated with DAO deficiency, our findings may not be as critical as expected. However, if most of the population does not have AOC1 gene variants related to DAO deficiency, our results may suggest that the DAO enzyme may play a critical role in the pathophysiology of ADHD. Finally, our results were based on reports of the parents that may be subject to recall bias of either the medical antecedents or comorbidities as assessed with Schnedl et al.’s questionnaire [25].

Longitudinal studies with bigger sample sizes are warranted to confirm our preliminary results and to explore possible causal relationships. Moreover, the correlations between genetic profiles and cognitive skills must be further compared with other patient populations. Future research is needed to fully understand the relationship between histamine, the DAO enzyme, and ADHD.

## Figures and Tables

**Table 1:** AOC1 gene variants studied and phenotypic interpretation.

Gen	Variant	Genotype	Phenotypic Interpretation
AOC1	c.47C>T (p.Thr16Met) (Variant 1)	CC CT TT	DAO normal functioning Mild DAO deficiency Severe DAO deficiency
	c.995C>T (p.Ser332Phe) (Variant 2)	CC CT TT	DAO normal functioning Mild DAO deficiency Severe DAO deficiency
	c.1990C>G (p.His664Asp) (Variant 3)	CC CG GG	DAO normal functioning Mild DAO deficiency Severe DAO deficiency
	c.-691G>T (Variant 4)	GG GT TT	DAO normal functioning Mild DAO deficiency Severe DAO deficiency

**Table 2:** Sociodemographic, clinical, and neuropsychological variables.

	Total (n = 303) %	Male (n = 219) %	Female (n = 84) %	*p* Value
Age: mean (sd)	13.2 (3.17)	13.17 (3.20)	13.29 (3.11)	0.766
Handedness: right	69	68.9	69	0.254
Ethnicity: Caucasian	86.5	86.8	85.7	0.740
ADHD, predominantly inattentive subtype (ADD)	29	25.6	38.1	0.098
Obesity	8.3	8.7	7.5	1
Atopy	63	62.9	63.3	1
Food allergy (any)	18.4	19.7	14.8	0.401
Drug allergy (any)	2.7	2.3	3.7	0.688
Traumatic injuries (any clinically relevant)	54.7	56.8	49.4	0.298
IQ (n = 174)	102.81 (20.13)	103.59 (19.27)	100.29 (22.76)	0.404
Verbal comprehension (n = 135)	103.33 (19.96)	104.88 (19.26)	98.74 (21.56)	0.146
Working memory (n = 121)	97.57 (17.59)	98.19 (16.61)	95.77 (20.35)	0.554
Processing speed (n = 123)	93.30 (14.56)	93.91 (15.01)	91.40 (13.13)	0.383
Perceptual reasoning (n = 69)	104.84 (20.08)	107.00 (19.70)	98.72 (20.44)	0.147

**Table 3:** Genotype variants and number of affected variants for gender.

Number of Positive (Dysfunctional) DAO Variants	Total (n = 303) %	Male (n = 219) %	Female (n = 84) %	*p* Value	Caucasian Only (n = 262) %	Other Ethnicities (n = 38) %	*p* Value
0	21.5	22.9	17.86	0.397	23.7	7.9	**0.002**
1	26.5	27.1	25		23.3	50.0	
2	27.5	26.6	29.8		28.6	18.4	
3	11.6	9.6	16.7		12.6	5.3	
4	12.9	13.8	10.7		11.8	18.4	
Variants							
Variant 1 (rs10156191)							
CC	50.2	53.4	41.7	0.226	50.0	52.6	0.617
CT	39.9	36.5	48.8		39.7	42.1	
TT	9.6	9.6	9.5		10.3	5.3	
Variant 2 (rs1049742)							
CC	81.5	81.3	82.1	0.761	82.4	78.9	0.709
CT	17.5	17.3	17.9		16.8	21.1	
TT	0.7	0.9	0		0.8	0	
Variant 3 (rs1049793)							
CC	43.8	47.5	48.8	0.580	51.1	28.9	**0.033**
CG	42.6	41.5	45.2		39.7	60.5	
GG	9.24	10.5	5.9		9.2	10.5	
Variant 4 (rs2052129)							
GG	51.2	51.6	50	0.901	50.4	60.5	0.486
GT	43.2	42.5)	45.2		44.3	34.2	
TT	5.3	5.48	4.76		5.3	5.3	

Bold is for significant *p* values.

**Table 4:** Genotype variants and number of affected variants for ADHD subtype.

	Total (n = 282) %	ADHD (n = 194) %	ADD (n = 88) %	*p* Value
Number of positive variants				
0	21.6	20.6	23.9	0.814
1	25.9	25.3	27.3	
2	27	26.8	27.3	
3	12.4	13.9	9.1	
4	13.1	13.4	12.5	
Variants				
Variant 1 (rs10156191)				
CC	49.6	47.9	53.4	0.530
CT	39.7	40.2	38.6	
TT	10.6	11.9	8	
Variant 2 (rs1049742)				
CC	81.2	81.4	80.7	0.774
CT	18.4	18	19.3	
TT	0.4	0.5	0	
Variant 3 (rs1049793)				
CC	48.9	47.4	52.3	0.681
CG	43.6	45.4	39.8	
GG	7.4	7.2	8	
Variant 4 (rs2052129)				
GG	50.7	49	54.5	0.449
GT	43.6	44.3	42	
TT	5.7	6.7	3.4	

**Table 5:** DAO genotype variants and intelligence and cognitive skills.

		Mean (sd) IQ (n = 174)	*p*	Mean (sd) Verbal Comprehension (VC) (n = 135)	*p*	Mean (sd) Working Memory (WM) (n = 121)	*p*	Mean (sd) Processing Speed (PS) (n = 123)	*p*	Mean (sd) Perceptual Reasoning (PR) (n = 69)	*p*
Variant 1 (rs10156191)	CC	101.82 (18.53)	0.465	101.12 (19.62)	0.185	**97.64 (17.09)**	**0.041**	93.53 (13.17)	0.709	106.42 (18.70)	0.365
	CT	105.04 (21.48)		107.22 (19.27)		**99.94 (17.29)**		93.98 (16.44)		105.27 (22.93)	
	TT	99.44 (23.00)		99.23 (23.98)		**85.18 (18.04)**		89.80 (12.99)		92.60 (5.90)	
Variant 2 (rs1049742)	CC	102.17 (20.04)	0.606	102.65 (19.35)	0.642	96.41 (17.38)	0.359	92.11 (14.05)	0.088	103.34 (19.11)	0.172
	CT	106.5 (21.74)		107.2 (24.16)		102.74 (18.59)		99.95 (16.05)		112.17 (24.39)	
	TT	103 (1.41)		106 (n/a)		97 (n/a)		95 (n/a)			
Variant 3 (rs1049793)	CC	102.14 (19.40)	0.531	101.41 (16.94)	0.380	95.08 (17.39)	0.251	92.82 (14.10)	0.857	101.89 (20.07)	0.371
	CG	104.81 (20.81)		106.43 (22.47)		100.76 (17.27)		94.33 (14.52)		109.23 (19.58)	
	GG	99.5 (21.84)		101.71 (24.55)		96.67 (19.19)		92.86 (17.49)		104.60 (24.98)	
Variant 4 (rs2052129)	GG	**99.26 (18.53)**	**0.002**	101.54 (19.21)	0.111	**96.19 (17.02)**	**0.016**	91.73 (12.82)	0.184	104.56 (19.36)	0.175
	GT	**108.51 (21.39)**		106.95 (21.23)		**101.16 (17.98)**		96.10 (16.83)		108.15 (22.09)	
	TT	**90.82 (11.37)**		93.38 (13.42)		**81.71 (8.73)**		88.17 (5.64)		89.80 (6.57)	

Bold is for significant *p* values.

**Table 6:** DAO variant and reduction severity related to cognitive skills.

		Mean (sd) IQ	*p*	Mean (sd) VC	*p*	Mean (sd) WM	*p*	Mean (sd) PS	*P*	Mean (sd) PR	*p*
Number of variants associated with DAO deficiency	0 (n = 65)	97.36 (15.33)	0.094	97.22 (15.70)	0.085	94.41 (15.29)	0.171	91.71 (10.89)	0.195	98.47 (15.41)	0.458
	1 (n = 80)	105.76 (21.28)		106.00 (22.06)		99.67 (18.53)		94.48 (15.48)		113.73 (22.52)	
	2 (n = 83)	103.07 (20.14)		104.69 (16.28)		94.92 (18.27)		91.81 (14.73)		101.38 (17.98)	
	3 (n = 35)	102.15 (26.44)		99.06 (27.60)		99.92 (19.06)		90.00 (15.90)		108.71 (22.65)	
	4 (n = 39)	109.00 (18.97)		113.08 (22.03)		105.00 (16.08)		102.29 (15.66)		107.88 (25.79)	
Phenotypic interpretation	Normal (n = 66)	**97.37 (15.33)**	**0.020**	**97.22 (15.70)**	**0.023**	94.41 (15.29)	0.252	91.71 (10.89)	0.399	98.47 (15.41)	0.081
	Reduced (n = 236)	**104.54 (21.23)**		**105.27 (20.91)**		98.37 (18.22)		93.93 (15.51)		107.04 (21.30)	
Severity of affectation of the DAO deficiency	Normal (n = 66)	97.37 (15.33)	0.612	97.22 (15.70)	0.71	94.41 (15.29)	0.571	91.71 (10.89)	0.830	98.47 (15.41)	0.839
	Mild Reduction (n = 187)	106.45 (20.82)		107.62 (20.12)		100.79 (17.92)		95.00 (15.65)		109.71 (21.39)	
	Severe Reduction * (n = 49)	98.56 (21.72)		97.22 (22.00)		89.21 (16.80)		89.95 (14.67)		96.10 (17.90)	

* Severe reduction meant that at least in one of the gene variants studied, the patient was homozygous for the defective alleles. Bold is for significant *p* values.

**Table 7:** Cronbach’s alpha for factor.

Factor	Number of Items	Cronbach’s Alpha
Gastrointestinal	9	0.703
Dermatological	4	0.687
Cardiovascular	5	0.568
Respiratory	3	0.810

## Data Availability

The data presented in this study are available on request from the corresponding author. The data are not publicly available due to confidentiality issues.

## Supplementary Materials

The following supporting information can be downloaded at: https://www.mdpi.com/article/10.3390/jcm13061659/s1, Table S1: Summary of significant results from Figure 1. Table S2: Summary of significant results from Figure 1 (cont.). Table S3: Summary of significant results from Figure 1 (cont.).
