# Supplementary material for: Prevalence and Clinical Picture of Diamine Oxidase Gene Variants in Children and Adolescents with Attention Deficit Hyperactivity Disorder: A Pilot Study
Source: J Clin Med. 2024 Mar 14;13(6):1659. doi: 10.3390/jcm13061659 (PMC10970994; doi:10.3390/jcm13061659)
Supplement: Supplementary file 1 [file jcm-13-01659-s001.zip › jcm-2830791-supplementary.pdf]

Table S1: Summary of significant results from Figure 1

		Variant 1			Variant 2			Variant 3			Variant 4		
		Normal	Mild reduction	Severe reduction	Normal	Mild reduction	Severe reduction	Normal	Mild reduction	Severe reduction	Normal	Mild reduction	Severe reduction
Schnedl et al.' test													
Dysmenorrhea	Yes	3 (2.0%)	8 (6.7%)	0									
	No	144 (98.0%)	111 (93.3%)	29 (100%)									
	Chi-square (p value)	5.261 (p = 0.072)											
Constipation	Yes							20 (13.8%)	30 (22.9%)	2 (9.5%)			
	No							125 (86.2%)	101 (77.1%)	19 (90.5%)			
	Chi-square (p value)							4.950 (0.084)					
Intestinal colic	Yes							4 (2.8%)	12 (9.2%)	1 (4.8%)			
	No							141 (97.2%)	119 (90.8%)	20 (95.2%)			
	Chi-square (p value)							5.265 (0.072)					
Itching	Yes							35 (24.1%)	31 (23.7%)	10 (47.6%)			

	No							110 (75.9%)	100 (76.3%)	11 (52.4%)			
	Chi-square (p value)							5.768 (p = 0.056)					
Palpitations	Yes										16 (10.5%)	27 (20.9%)	2 (12.5%)
	No										136 (89.5%)	102 (79.1%)	14 (87.5%)
	Chi-square (p value)										5.968 (p = 0.051)		
Medical antecedents													
Lung disorders	Yes							5 (3.4%)	0	0			
	No							140 (96.6%)	132 (100%)	20 (100%)			
	Chi-square (p value)							5.331 (p = 0.070)					
Thyroid disorders	Yes							1 (0.7%)	0	1 (5.0%)	1 (0.7%)	0	1 (6.2%)
	No							144 (99.3%)	132 (100%)	19 (95.0%)	151 (99.3%)	129 (100%)	15 (93.8%)
	Chi-square (p value)							6.493 (p = 0.039)					
Kidney disorders	Yes										0	1 (0.8%)	1 (6.2%)

	No										152 (100%)	128 (99.2%)	15 (93.8%)
	Chi-square (p value)										8.490 (p = 0.014)		
Binge eating disorder	Yes				13 (5.37%)	9 (17.65%)	0						
	No				229 (94.63%)	42 (82.35%)	2 (100%)						
	Chi-square (p value)				9.359 (p = 0.009)								
Glasses	Yes										48 (31,37%)	29 (23.39%)	8 (50%)
	No										105 (68.63%)	95 (76.61%)	8 (50%)
	Chi-square (p value)										5.741 (p = 0.057)		

Table S2: Summary of significant results from Figure 1 (cont.)

		Variant 1		Variant 2		Variant 3		Variant 4	
		Normal	Reduced	Normal	Reduced	Normal	Reduced	Normal	Reduced
Schnedl et al.' test									
Vertigo	Yes	8 (5.4%)	17 (11.4%)						
	No	140 (94.6%)	132 (88.6%)						
	Chi-square (p value)	3.472 (0.062)							
Intestinal colic	Yes					4 (2.8%)	13 (8.6%)		
	No					141 (97.2%)	139 (91.4%)		
	Chi-square (p value)								
Postprandial satiety	Yes							29 (19.2%)	17 (11.7%)
	No							122 (80.8%)	128 (88.3%)
	Chi-square (p value)							3.154 (p = 0.076)	
Palpitations	Yes							16 (10.5%)	29 (20.0%)
	No							136 (89.5%)	116 (80.0%)
	Chi-square (p value)							5.181 (p = 0.024)	
Medical antecedents									
Kidney disorders	Yes					5 (3.4%)	0		
	No					140 (96.6%)	152 (100%)		

	Chi-square (p value)					5.331 (p = 0.021)			
Migraines	Yes					10 (6.9%)	20 (13.2%)		
	No					135 (93.1%)	132 (86.8%)		
	Chi-square (p value)					3.204 (p = 0.073)			

Table S3: Summary of significant results from Figure 1 (cont.)

		Hypotonia	p	Itching	p	Eczemas or eruptions	p
Variant 1 (rs10156191)	CC	15 (10.13%)	0.032				
	CT	19 (15.83%)					
	TT	5 (17.24%)					
Variant 2 (rs1049742)	CC	32 (13.22%)	0.080				
	CT	7 (13.20%)					
	TT	0					
Variant 3 (rs1049793)	CC	15 (10.59%)	0.043	35 (24,47%)	0.037	42 (29.37%)	0.029
	CG	20 (15.75%)		28 (22.05%)		34 (26.67%)	
	GG	4 (14.81%)		13 22.81%)		14 (51.85%)	
Variant 4 (rs2052129)	GG	15 (9.87%)	0.047				
	GT	21 (16.28%)					
	TT	3 (18.75%)					
